# *Journal of Otolaryngology Head & Neck Surgery* – An ‘Open’ Letter to Our Authors and Readers

**DOI:** 10.1186/1916-0216-42-1

**Published:** 2013-01-31

**Authors:** Hadi Seikaly, Erin D Wright

**Affiliations:** 1 Department of Surgery, Division of Otolaryngology-Head and Neck Surgery, University of Alberta, Edmonton, AB, Canada; 2 Division of Otolaryngology-Head and Neck Surgery, University of Alberta, Edmonton, AB, Canada

## 

The *Journal of Otolaryngology-Head & Neck Surgery* is at a significant threshold. As a mature, respected, and long-lived entity it has grown from the scientific publication of a small national society (Canadian Journal of Otolaryngology, 1971) to a major international journal (Journal of Otolaryngology-Head & Neck Surgery, 2008). The process of development has resulted in the evolution of the Journal from paper based manuscript management to an online submission system, and we are now advancing to a completely digital, open access format effective January 2013.

The reasons for the change are several. Open access publishing has advantages that include universal, free, and immediate access to the published content in standard repositories (e.g. PubMed). The single biggest advantage of open access publishing, as it pertains to the Journal, is the potential to increase the impact factor by virtue of higher downloads and citations [1,2]. Finally, open access publishing is in full compliance with the true intent of major funding bodies such as the CIHR, NIH, Wellcome Trust, and Howard Hughes Medical Institute [3-7], which require open access to funded research within 12 months of publication, and grants from these entities can be used to cover the costs of open access publication. Open access publishing of scientific material has been commonplace in the basic sciences for some time and we are certain that medical sciences will shortly follow suit. We are the first PubMed indexed and Thomson-Reuters tracked (i.e. with an Impact Factor) journal in the field of Otolaryngology-Head & Neck Surgery to enter into this publishing model and are poised to reap the rewards of the advantages noted above by being an early adopter.

We have chosen to publish with BioMed Central, a pioneer in open access publishing, based on their experience with this model of publishing, substantive logistical support, stringent peer review process, as well as their commitment to a sustainable model from scientific, financial, and environmental perspectives. As such, BioMed Central has committed to permanent accessibility of its content both past and present and has taken measures to ensure that this remains the case in perpetuity.

In brief, the open access publishing model transfers the cost of publishing from the subscribers (societies, libraries, clinicians) to the researchers and funders and essentially makes publishing the final step in the research process, providing free and immediate access to published content. In so doing, the restricted access of the traditional subscription-based model is circumvented. This free and immediate access is permitted by the Article Processing Charge (APC), payable one time only as a flat fee, with no additional charges for colour figures, videos, or large amounts of data. In addition to the potential for research grants to cover the costs of APCs, many institutions have memberships in BioMed Central (http://www.biomedcentral.com/inst/), which will offset a portion of, or the entire cost of the APC. In the case of our Journal certain articles may, at the discretion of the editors, have the APC covered by the Canadian Society of Otolaryngology-Head & Neck Surgery.

As we proceed with the implementation of this new model we anticipate encountering some initial confusion, and possibly resistance, to this new model, particularly as it pertains to the costs of publishing. Fortunately, most of our authors are affiliated with institutions (mainly Universities) that have full or supporter memberships in BioMed Central and are thus eligible for the aforementioned reductions or waiver of the APC. In addition, in order to assist authors from developing and less economically privileged countries, BioMed Central operates a waiver program [8], which can be accessed or requested at the time of article submission.

There are several advantages that we anticipate realizing as a result of the transition to open access publishing. It is our hope that basic science and clinical research that is funded by national granting agencies will see this as a superior vehicle for dissemination of their research products, thus increasing the content of this peer review funded scholarship in our Journal. In addition, as a result of the increased accessibility and visibility, we fully anticipate an increase in our impact factor in the years to come.

In terms of content, process, and policy at the Journal we do not anticipate substantive changes. Our Editorship (Drs. Seikaly and Wright) and Managing Editor (Ms. Stacey Leavitt-Wright) as well as our Associate Editors (http://www.journalotohns.com) remain unchanged from previously. With some slight changes related to different site structure our authors and reviewers will find the peer review logistics to be quite similar. Our peer review process will remain stringent but fair and as constructive as possible. The one change that authors and readers may note is related to one of the advantages of online only open access publishing and that is a faster, more streamlined review and, most importantly publishing process with almost immediate publication of accepted material (once formatting and typesetting has been completed).

To conclude, the Editors and Editorial Board are enthusiastic about our new publishing model and the potential advantages that it affords. Changes and transitions are, of course, times to explore new opportunities and we are contemplating new areas of focus and process once we have successfully solidified the new publishing model. As always we remain indebted to our Society, authors Associate Editors and peer reviewers, without whose hard work, dedication, attention to detail, we could not have reached this point nor contemplated a robust and successful future. We look forward to continuing these strong relationships and to working with our new Publisher.
